# A Rapidly Expanding Neck Mass with Risk of Airway Compromise: A Case Report

**DOI:** 10.5070/M5.52999

**Published:** 2026-10-31

**Authors:** Hannah Sloan, Emmelyn J Samones, William Michael Downes

**Affiliations:** * Loma Linda University Health, School of Medicine, Loma Linda, CA; ^ Loma Linda University Medical Center, Department of Emergency Medicine, Loma Linda, CA

## Abstract

Airway compromise and impending collapse should be considered in all patients presenting with a neck mass. This patient presented to the emergency department (ED) two weeks prior with concerns for rapid growth of a neck mass; after being sent home with an outpatient referral to specialty services, she re-presented when she developed progressive dyspnea and stridor. Imaging was concerning for anaplastic thyroid carcinoma that had grown over two centimeters (cm) in the past 15 days from 10.1 cm to 12.2 cm with new tracheal deviation and adjacent thrombus compression of the internal jugular vein and superior vena cava. Emergency medicine physicians should be cautious when discharging a patient with a neck mass. Outpatient referral should be considered only when the stability of the size of the mass can be reliably confirmed and close outpatient follow-up is guaranteed. Without these stipulations, ED physicians should advocate for admission of these patients so that specialty teams, such as otolaryngology, interventional radiology, and hematology/oncology, can begin management before airway compromise poses a significant threat to the life of the patient.

**Topics:**

Neck mass, thyroid cancer, risk of airway compromise with mass effect.

**Figure:**
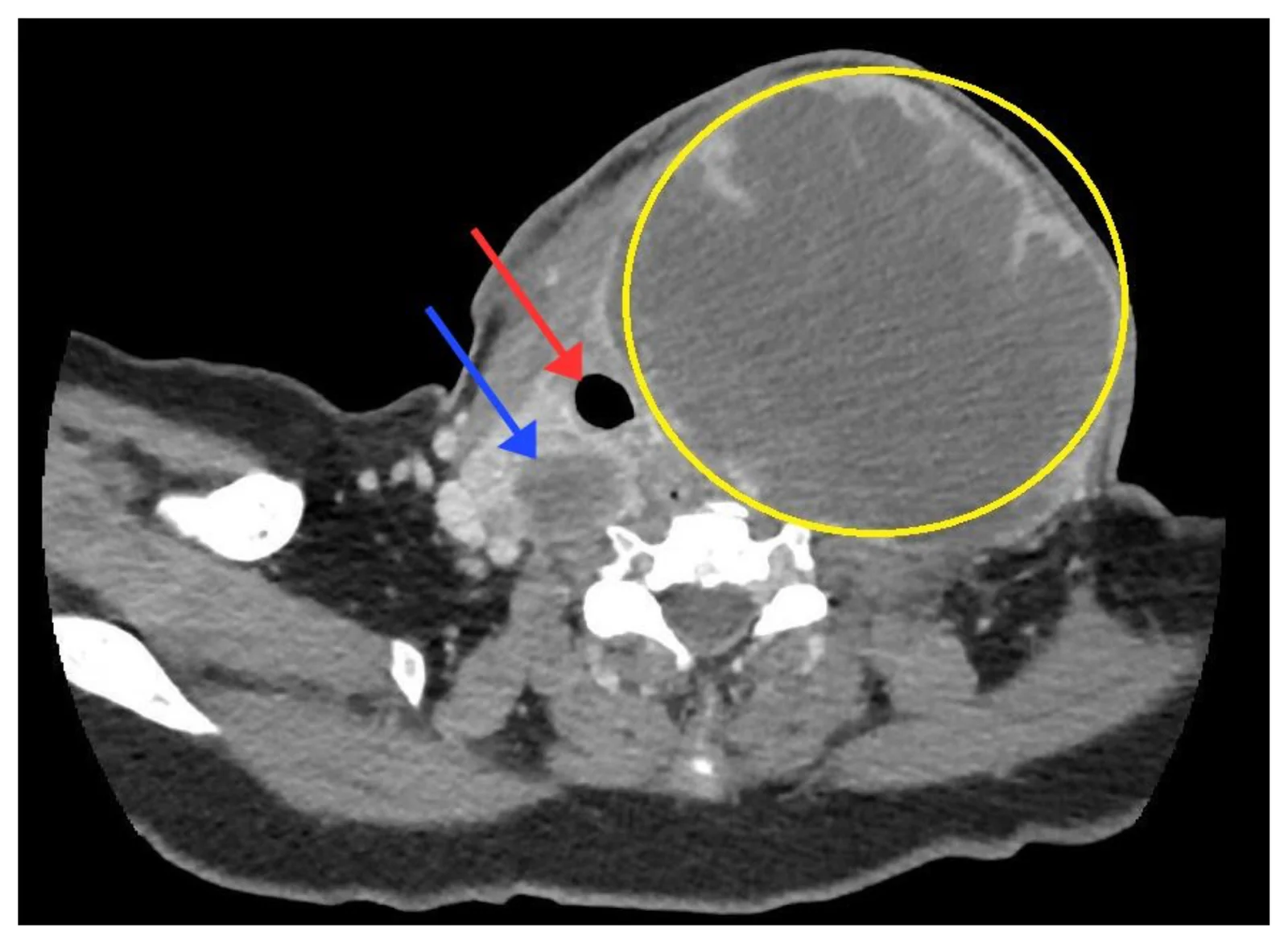


**Figure:**
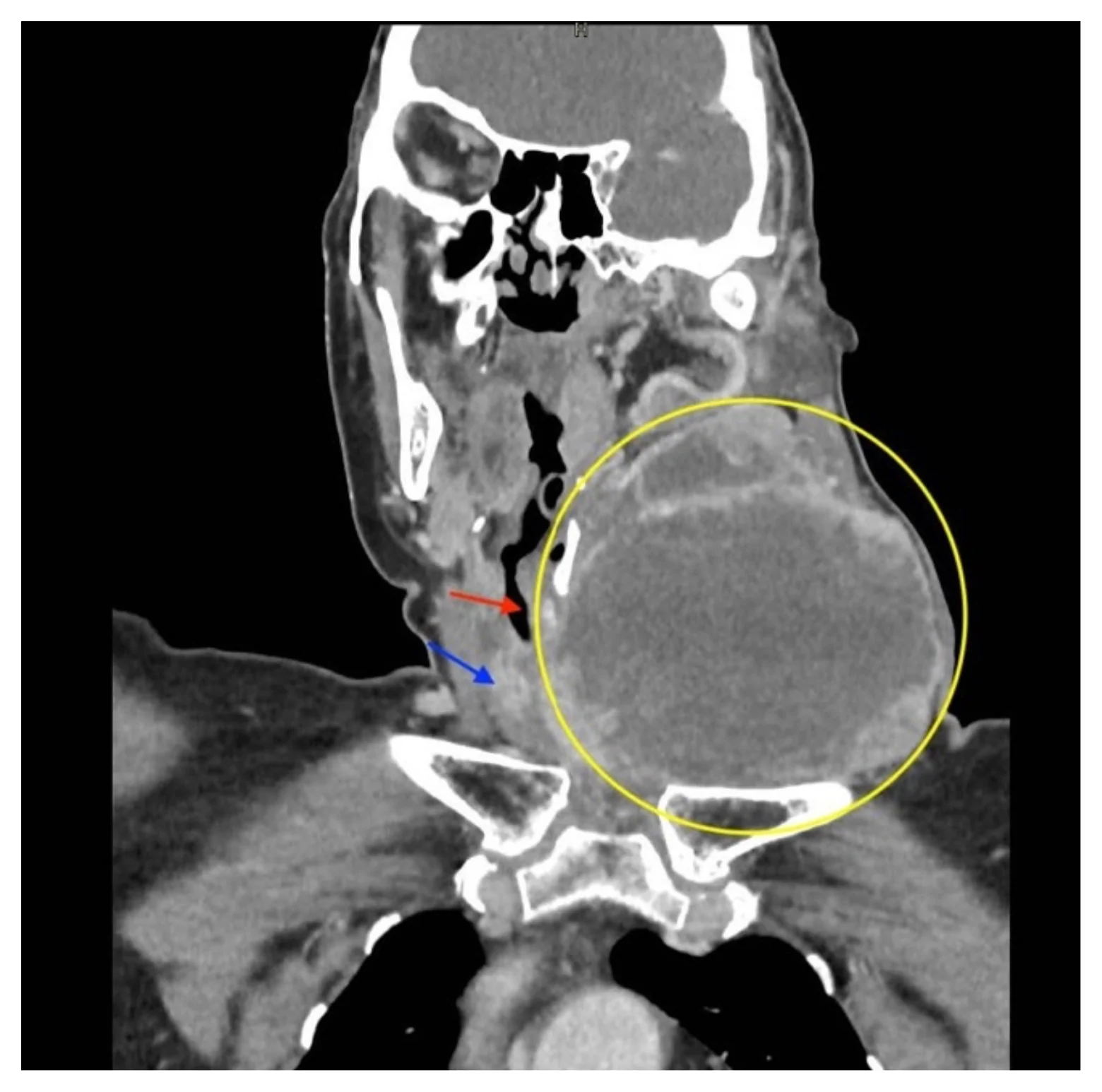


**Figure:**
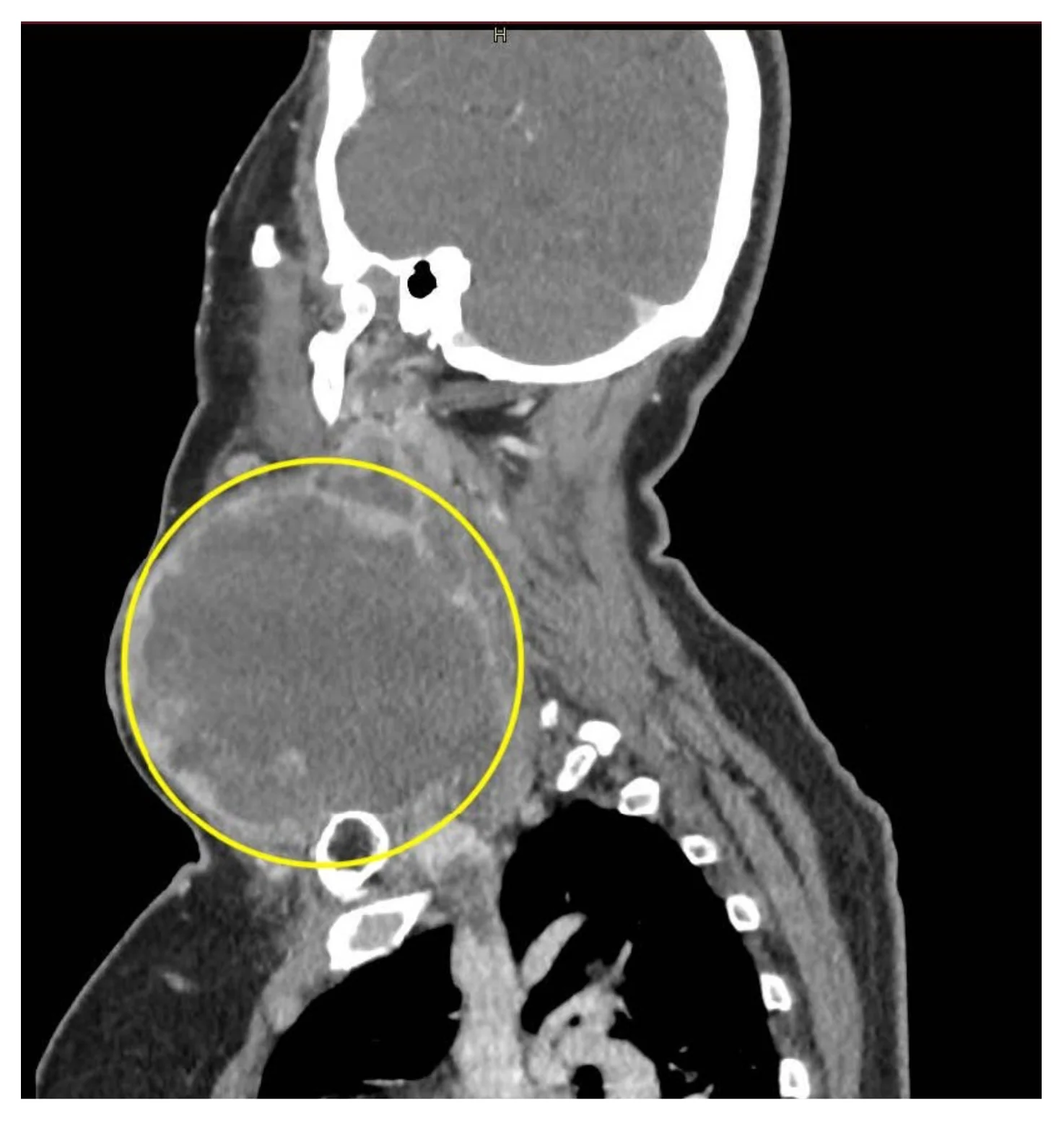


## Brief introduction

Neck masses pose a significant threat to the natural airway of our patients. There are many etiologies and various causes of neck masses. Our focus is on thyroid masses, which are present in a much higher incidence in women.^1^–^2^ Anaplastic thyroid carcinoma is a lethal type of neck mass, primarily due to its rapid growth and inherent risk for airway compromise.^1^,^3^ Clinicians should be wary for this presentation, and significant consideration should be taken in patients who present with a lateral neck mass of at least five centimeters with reported rapid increase in size.^1^ According to the American Thyroid Association, “significant growth of a thyroid nodule is considered when the nodule grows 20% in at least two dimensions with an increase of two millimeters (mm) or more or when the nodule increases by 50% or more in volume in a year.”^4^–^5^ Due to various reasons, this type of cancer can rarely be cured; because of this, physicians should be focused on preventing death in these patients from internal asphyxiation and suffocation.^6^

## Presenting concerns and clinical findings

A 66-year-old female with a past medical history of thyroid mass with prior fine needle aspiration (FNA) and left thyroidectomy presented to the emergency department (ED) with increasing neck pain and increased size of known neck mass. The patient previously presented to the ED two weeks prior with similar concerns. During that visit, she was evaluated by ear, nose, and throat (ENT) service who did not have concerns for immediate airway compromise and recommended a STAT referral to outpatient hematology and oncology for further management with strict precautions to return if she developed dysphagia or difficulty breathing. At the time of discharge, the patient did not have stridor, was tolerating secretions, and was saturating at 99% on room air without increased work of breathing. She was then, however, unable to obtain a close follow-up appointment, and she re-presented to the ED due to noticing the neck mass had doubled in size with new difficulty breathing and chest pain. The patient stated that the mass had been present for about seven years, but in the last month had a rapid increase in growth, from the initial size of about a marble. The patient had biopsies performed four months prior, and had a resection in another country; the results had come back with concern for anaplastic carcinoma. Since resection, the neck mass had recurred and was now larger than ever before. In the ED, she was found to be afebrile 97.7 degrees Fahrenheit, blood pressure of 151/94 milligrams of mercury, heart rate of 87 beats per minute, respiratory rate of 24 breaths per minute, and SpO2 98% on room air. On physical exam she was found to have a large left-sided neck mass and audible stridor but was still protecting her airway.

## Significant findings

Computed tomography (CT) scan of the neck soft tissue with contrast 15 days prior to current presentation demonstrated a centrally necrotic left thyroid mass measuring 10.1 cm and adjacent tumor thrombus within the adjacent left internal jugular vein extending superiorly to level of C1 without intracranial extension. There was regional mass effect with associated mild narrowing of the left common carotid artery and multiple additional lesions in right thyroid lobe, largest 2.9 cm. There was a necrotic left lower cervical lymph node of 1.1 cm with concern for nodal metastasis.

Repeat CT scan soft tissue neck with contrast was obtained in the ED, which showed rapid enlargement of a large centrally necrotic left thyroid mass compatible with malignancy (yellow circle) measuring up to 12.2 cm (previously 10.1 cm 15 days prior), increased overall clot burden, and extension of adjacent tumor thrombus within the left internal jugular vein extending superiorly to level of C1 and inferiorly into duplicated left superior vena cava without intracranial extension. Imaging also revealed worsening regional mass effect on the adjacent structures with increased compression and partial effacement of the extra-thoracic trachea (red arrow) with rightward tracheal deviation and partial encasement, similar to slightly worsened narrowing of the left common carotid artery. Imaging also revealed increased size of additional lesions in right thyroid lobe (blue arrow) measuring 3.5 cm (previously 2.9 cm 15 days prior).

## Patient course

This patient had originally presented to the ED 15 days prior. At that time, she was evaluated by ENT with no concern for immediate airway compromise, and the patient was discharged with a STAT referral for an appointment with hematology/oncology and strict return precautions. In the 15-day interim, the patient, however, was not able to make an appointment with the oncology team, which led to her return to the ED. At the time of this encounter, the mass had grown over 2 cm, and the thrombus had now extended into the internal jugular vein; additional mass effect was also causing significant tracheal deviation. On assessment, the patient had stridor, which was extremely concerning for airway collapse; however, the patient was found to be protecting her airway appropriately. Ears, nose, and throat service was then consulted for further management due to concern for difficult airway. ENT was most concerned for a rapidly expanding anaplastic carcinoma; however, they did not find evidence of immediate airway compromise. They presented two options to the patient for further management because they believed, due to the compressive effects of the mass, she did not have enough time to wait for an outpatient appointment with oncology. They informed the patient her options were to be admitted to the hospital for initiation of targeted radiation therapy or to place a palliative tracheostomy. The patient opted for admission to medicine with an inpatient consult to hematology/oncology and initiation of targeted radiation therapy. Interventional radiology (IR) was then consulted for mapping and planning of radiation therapy. The patient was subsequently admitted to the inpatient medicine team, started on high dose steroids, and mapping was completed for radiation therapy with subjective decrease in mass size and relief of her symptoms. She was discharged to follow up with hematology/oncology. The patient attended a few hematology/oncology appointments; however, the remainder of her treatment course is unknown because she was lost to follow-up.

## Discussion

This patient’s case presented several challenges for the ED team. Although a STAT referral to hematology/oncology is reasonable, there are many factors to consider the utilization of inpatient admission. More recently, the time patients must wait to see an outpatient specialty service has increased. When clinicians discharge patients from the ED, they have limited ability to ensure that referral is followed up on in a reasonable amount of time. Additionally, the patient was concerned that her mass had been rapidly growing over the course of the last month. Understanding that significant growth of a thyroid mass is considered to be an increase of two millimeters over a year, a one-month noticeable increase in size by the patient is concerning. This prompt growth should alert ED physicians to the risks of impending airway compromise with any neck mass, and physicians should advocate for inpatient admission for prompt management by specialty teams such as ENT, IR, and hematology/oncology, rather than outpatient evaluation because time may be of the essence.

At this presentation, the patient arrived at the ED with concerning signs and symptoms of airway compromise. This was difficult because the patient did not have an optimal option for an emergent airway. If the patient declined rapidly, direct laryngoscopy would have presented a significant challenge. Because of the mass effect, the patient also had severely limited ability to open her mouth, tracheal deviation, and approximately 50% collapse of the proximal trachea. All of these factors would prove a significant challenge in the event of an emergent need for airway. These findings should prompt ED physicians to consult ENT emergently and when possible, hold off on placing an airway. In this case when ENT was consulted, it was recommended if an emergent airway was needed, that included attempting a small endotracheal tube or a fiberoptic approach to intubation.

Physicians should consider when to offer a palliative tracheostomy emergently in patients with neck masses that are of significant risk to airway patency. The current recommendations by the American Thyroid Association suggest avoiding elective tracheostomy and only considering an operative tracheostomy in the emergency setting when airway compromise is definitive.^3^ These tracheostomies should not be done in the ED and should be placed surgically when needed.^2^,^3^ Thus, in patients like our case study, as long as the patient is appropriately protecting their airway, tracheostomy should be avoided in the emergency setting.^3^,^7^ Close monitoring of these patients is vital and continued reassessment is needed to ensure the airway continues to be patent.

## Supplementary Information












